# Clinical and genetic profile in index patients with spinocerebellar ataxia type 3 in Indonesia: case report

**DOI:** 10.1016/j.heliyon.2021.e07519

**Published:** 2021-07-07

**Authors:** Siti Aminah, Fathul Huda, Uni Gamayani, Iin Pusparini, Mochammad Faisal Afif Mochyadin, Yunia Sribudiani, Norlinah Mohamed Ibrahim, Tri Hanggono Achmad

**Affiliations:** a Department of Neurology, Faculty of Medicine, Universitas Padjadjaran / Dr. Hasan Sadikin Central General Hospital, Bandung, Indonesia; b Research Center of Medical Genetics, Faculty of Medicine, Universitas Padjadjaran, Bandung, Indonesia; c Department of Biomedical Sciences, Division of Physiology, Faculty of Medicine, Universitas Padjadjaran, Bandung, Indonesia; d Department of Biomedical Sciences, Division of Biochemistry and Molecular Biology, Faculty of Medicine, Universitas Padjadjaran, Bandung, Indonesia; e Department of Medicine, Faculty of Medicine, Universiti Kebangsaan Malaysia Medical Center, Kuala Lumpur, Malaysia

**Keywords:** Autosomal dominant, Familial ataxia, Indonesia, Polyglutamine, Spinocerebellar ataxia

## Abstract

Spinocerebellar ataxia (SCA) is an autosomal dominant hereditary disease with progressive course, and no causal therapy. Diagnostics are still challenging, due to facility and protocols, and so as in Indonesia. As a national referral center, Dr. Hasan Sadikin Central General Hospital has received a lot of patients from all over Indonesia, particularly from Western Java. Study related to SCA (including clinical and genetic profile) is still limited in Indonesia. We identified index patients from three families with ataxia, hence intend to determine their clinical and genetic pattern.

The hereditary pattern is autosomal dominant. Scale for the assessment and rating of ataxia (SARA) shows mild and moderate ataxia. Inventory of non-ataxia signs (INAS) scores of the patients were 3, 5 and 6. Montreal cognitive assessment-Indonesian version (MOCA-INA) shows only one patient has mild cognitive impairment, despite young age. Barthel index shows 1 subject has moderate dependency. Mutation in Ataxin3 polyQ repeats shows pathologically long CAG repeats, 72,10; 72,10; and 72,23 respectively in mutant and wild type allele.

We diagnosed the index patients with spinocerebellar ataxia type 3. This study is the first case series study in Indonesia. The hereditary pattern is clearly shown as an autosomal dominant ataxia. The clinical and genetic profile was varied, and the symptom is progressive and deteriorates overtime, including wide based gait, speech problem, motor and sensor complaint, and cognitive decline complaint. Despite the same polyQ stretch length, the onset and clinical characteristics of patients are diverse.

## 1 Introduction

Spinocerebellar ataxia (SCA) is an autosomal dominant hereditary degenerative disease that has no causal therapy. The prevalence is 1–5/100000 people, varied upon ethnicity and geographic [1].

Among all 47 types of spinocerebellar ataxia that have been identified, type 1, 2, 3, 6 and 7 are the most common in the world, with type 3 being the most prevalent. SCA is a heterogeneous disease, with complex phenotype and genetics. The degree of clinical symptom related to its anatomy involvement varied from being ataxia only, gait problem, visual problem, dysarthria, dysphagia, cognition problem and motor-sensory problem to name a few. The course of the disease is clearly progressive, with mortality in later stage due to progression which lead to immobility and inability to breathe [1, 2, 3, 4, 5, 6]. Up until now only supportive therapy is available, with recent advances in therapeutic approaches related to stem cell and genetic intervention [7]. The most common pathology of SCA is an autosomal dominant with a long polyglutamine (CAG) repeat in coding region of a gene and other repeat sequences in non-coding region. Spinocerebellar Ataxia 3 (SCA3) is caused by CAG repeat expansion (polyQ) in *SCA3* (a.k.a *ATXN3*) gene. The length of CAG repeat in wild type *ATXN3* is less than 40, and in a patient with SCA the repeat would be more than 40 [1,8]. The main mechanisms of dysfunction due to polyQ are caused by misfolding of mutant polyQ protein into inclusion body via beta-sheet monomer and oligomers. This misfolded protein in soluble form and moreover in inclusion body form are toxic in nature, disrupting neuron communication well-being, and then arose as clinical symptoms via toxic gain, protein loss of function, mitochondrial dysfunction, channelopathy, autophagy and transcription dysregulation [9]. There is also a phenomenon called anticipation, where the descendant has more repeats than the parents. The longer the repeats the earlier the onset and the worse the severity. Clinical and genetic profile is varied between individuals, even in the same family. Hence a variety of disease onset, progression and severity might be found in the same family with the same pathology [1, 7, 8, 10].

Diagnostics in developing countries are still a challenging endeavor, due to facility and protocols, and so as in Indonesia. The standard protocols are including clinical finding of ataxia and related sign and symptom, exclusion of a direct or secondary ataxia, a positive family history, and specific gene testing [7]. With limited doctor to patient ratio, and genetic facility, moreover a rather insensitivity or disregarding illness from the patients, a more prudent and comprehensive approach is necessary [5, 7, 11, 12].

In Indonesia, epidemiology and genetic study of SCA, is not yet well established. Indonesia is a huge country, geographically and genetically. With more than 1.9 million sq. km area, more than 260 million inhabitants, 17,000 islands and more than 1,000 ethnicity, medical care is an enormous challenge. As a national referral center, Dr. Hasan Sadikin Central General Hospital has received a lot of patients from all over Indonesia, particularly from Western Java, as the highest populated province, with almost 45 million inhabitants. From all patients with ataxia in 2020, we identified index patients from three families with ataxia, and we determined their clinical and genetic pattern.

### 1.1 Patient information

This study investigated three index patients from three different families with progressive ataxia, that also appears in other close relative members, from different cities in West Java Province. Patients were de-identified to protect their privacy, without hindering analysis of clinical information. Before come to our center, the patient had limited prior medical evaluation exposure, moreover genetic evaluation.

### 1.2 Clinical findings

**Figure 1:**
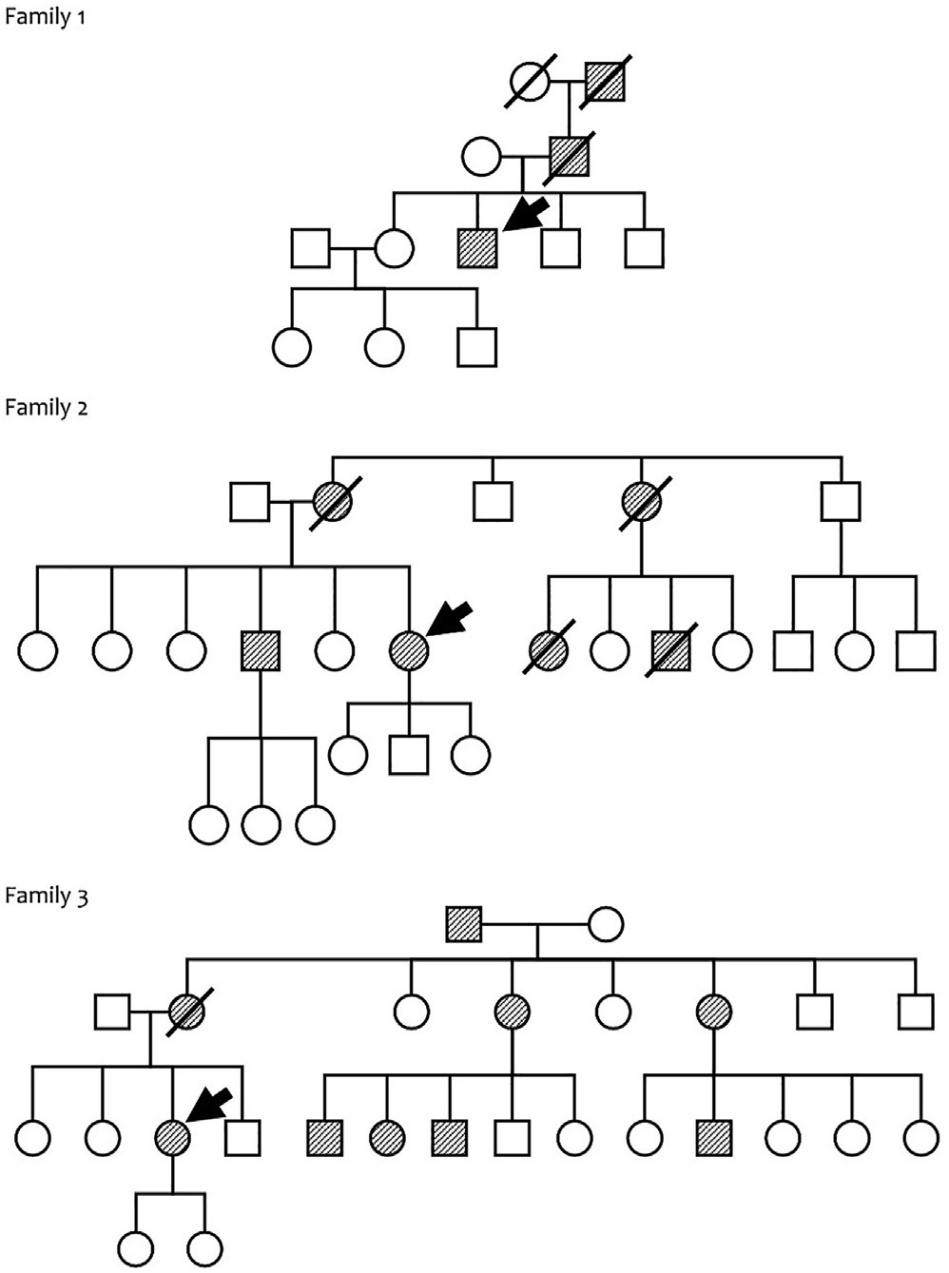
The pedigree of those 3 families shows an autosomal dominant pattern of inheritance, without generation skip and involving both male and female. Index patient in each family is shown by arrow.

Three index patients in their 30s from different families came to us with a chief complaint of unsteady walking and gait problems, with similar history in other family members. History, physical and neurological examination, INAS, SARA, MOCA-INA, Barthel Index, pedigree analysis, and SCA panel analysis were done for those index patients. Pedigree analysis was done, history was taken from the subject and digging the family history for ataxia complaint in the family (Figure 1). Patients provided written informed consent to the study (Table 1).

**Table 1:** Profile and Clinical finding of the index patients.

		Patient #1	Patient #2	Patient #3
**Sex**		M	F	F
Age (year-old)		32	38	32
Age at onset (year-old)		18	14	27
Evolution(years)		14	24	5
**Education**		high school	university	university

**Family with symptoms**		Yes	Yes	Yes
	**Number of family members**	12	24	26
	**Generation**	4	3	3
	**Family member with clinical ataxia**	3	6	9
**First complaint**		speech problem (14 years)	unsteady walk (24 years)	unsteady walk (5 years)

**Brain involvement**	**Cerebellum**	slurred and scanning speech (14 years), unsteady walk (2 years).	unsteady walk (24 years), dizziness (20 years), speech problem (5 year)	unsteady walking (5 years)
	**Brain Stem**	choking (3 years), dizziness (2 years),	double vision (4 years), choking (1 year)	swallowing difficulties & choking (3 years), dizziness (3 years)
	**Cerebrum**	(-)	difficulties in writing (20 years), involuntary hand movement (20 years)	vognitive problem (3 years)
	**Sensory**	left foot numbness (3 years)	(-)	no disturbance
**Clinical examination**	**Vital Sign**	no disturbance	no disturbance	no disturbance
	**Cranial Nerve**	rotational Nystagmus, dysphagia	dysphagia, Tongue fasciculation	horizontal nystagmus, left NVII weakness, dysphagia, tongue atrophy + fasciculation
	**Motor**	no weakness	difficulties in writing (20 years), dystonia (20 years)	mild weakness of extremities
	**Muscle tone**	increased	increased	increased
	**Sensory**	left foot numbness	(-)	(-)
	**Gait**	(-)	wide based gait	wide based gait
	**Coordination**	disturbed	disturbed	disturbed
	**Physiological Reflex**	BTR, KPR, APR + 3/+3	BTR, KPR, APR + 3/+3	BTR, KPR, APR + 3/+3
**Assessment Tools**	**MOCA INA**	28	30	24
	**INAS**	3	5	6
	**SARA**	4	9	14
	**Barthel**	100	80	100

MOCA INA: Montreal cognitive assessment Indonesian version, INAS: Inventory of Non-Ataxia Sign, SARA: Scale for the assessment and Rating of Ataxia.

The symptoms are progressing by age, starting from their 20s for patient #1 and #3 and in teenage for patient #2. The symptoms accompanied or followed by slurred and scanning speech, dizziness, choking, numbness, and nystagmus in patient #1; dizziness, motor problem, speech problem, and choking in patient #2. In patient #3 it was followed by choking, spinning dizziness, cognitive problem, horizontal nystagmus, left NVII palsy, tongue atrophy, and paresis. All patients showed disturbed coordination and increased physiological reflexes.

The patients have graduated from high school and university, this proves that they have good cognition to begin with to finish their education.

The history, physical and neurological examination are presented in Table 1.

### 1.3 Scale for the assessment and rating of ataxia (SARA)

To show the severity of ataxia in these index patients, SARA was utilized. SARA of these patients show all patients have ataxia in different categories. SARA scores were 4, 9 and 14 which means categorized as mild ataxia, mild ataxia and moderate ataxia, respectively, as shown in Table 2.

**Table 2:** Scale for the Assessment and Rating of Ataxia in the index patient.

Domain		Patient #1	Patient #2	Patient #3
Gait		**1**	**3**	**3**
Stance		**1**	**3**	**3**
Sitting		**0**	**0**	**0**
Speech disturbance		**1**	**1**	**0**

Finger chase	*R*	*0*	*0*	*2*
	*L*	*1*	*2*	*2*
	**Mean**	**0,5**	**1**	**2**
Nose-finger test	*R*	*0*	*0*	*2*
	*L*	*1*	*0*	*2*
	**Mean**	**0,5**	**0**	**2**
Fast alternating hand movements	*R*	*0*	*0*	*1*
	*L*	*0*	*0*	*1*
	**Mean**	**0**	**0**	**1**
Heel-shin slide	*R*	*0*	*1*	*3*
	*L*	*0*	*1*	*3*
	**Mean**	**0**	**1**	**3**
**Total**		**4**	**9**	**14**

note, R: right, L: left. Mean is used for total score summation whenever right and left sides are examined.

### 1.4 Inventory of non-ataxia sign (INAS)

Lately, patients with SCA also showed and proven to have extracerebellar pathology involvement. INAS was used to provide structured information of non-ataxia signs in ataxia patients. This score can be used as a simple semi-quantitative variable for extracerebellar involvement in SCA. Scores for the patients were 3, 5 and 6, which gave clues that there are extracerebellar involvement in these patients. A detailed description of INAS domain is as shown in Table 3.

**Table 3:** Inventory of non-ataxia sign (INAS) of the index patient.

Domain	Patient #1	Patient #2	Patient #3
Areflexia	0	0	0
Hyperreflexia	1	1	1
Extensor plantar response	0	1	1
Spasticity	0	0	0
Paresis	0	1	1
Amyotrophy	0	0	0
Fasciculations	0	1	1
Myoclonus	0	0	0
Rigidity	0	0	0
Chorea	0	0	0
Dystonia	0	1	0
Resting tremor	0	0	0
Sensory symptoms	1	0	0
Brainstem oculomotor signs	1	0	1
Urinary dysfunction and	0	0	0
Cognitive impairment	0	0	1
**Total**	**3**	**5**	**6**

### 1.5 Barthel index

To provide a sensible daily independency from help, Barthel index was employed. In these index patients, only patient #2 showed a moderate dependency, while the rest was deemed to be able to live independently as depicted in Table 4.

**Table 4:** Barthel index of the index patient.

Barthel Index	Patient #1	Patient #2	Patient #3
Feeding	10	10	10
Bathing	5	5	5
Grooming	5	5	5
Dressing	10	5	10
Bowels	10	10	10
Bladder	10	10	10
Toilet Use	10	10	10
Transfer (bed to chair and back)	15	10	15
Mobility (on level surface)	15	10	15
Stairs	10	5	10
**Total**	**100**	**80**	**100**

### 1.6 Pedigree analysis

Pedigree analysis was done to evaluate the pattern of inheritance, the number of family members with symptoms, and the position of these index patients in their own respective family. After history taking and pedigree analysis it is known that the pattern of inheritance is an autosomal dominant in all three families. Family 1, consists of 12 family members from 4 generations, with 3 family members showing symptoms. Family 2, consists of 24 family members from 3 generations with 6 family members showing symptoms. Family 3, consists of 26 family members from 3 generations, with 9 family members showing symptoms (Figure 1).

### 1.7 SCA PolyQ repeat panel analysis

Access to genetic evaluation and facility is still scarce in Indonesia. Only certain facilities in certain cities able to do routine examinations. Hence lesser number of facilities are able to do development and establish new genetical evaluation protocol. Our facility is equipped with molecular genetic instruments that enable us for genetic evaluation and sequencing, which may be considered essential in an advanced facility, but scarce in developing countries. These instruments are enabling us to establish new protocols in diagnosing SCA and sequencing polyQ repeats.

We did a SCA polyQ repeat analysis, it showed that these 3 index patients have a mutation in *ATXN3* (SCA3) with pathologically long CAG repeats. The repeats in mutant and wild type allele were 72, 10; 72, 10; and 72, 23, respectively. The details were shown in Table 5.

**Table 5:** PolyQ repeat in both alleles of the index patients.

Gene	Band	Patient #1	Patient #2	Patient #3
*Ataxin3*	Mutant alelle	(CAG)_2_CAAAAG(CAG)CAA (CAG)_70_	(CAG)_2_CAAAAG(CAG)CAA(CAG)_70_	(CAG)_2_CAAAAG(CAG)CAA (CAG)_70_
	Wild type alelle	(CAG)_2_CAAAAG(CAG)CAA(CAG)_8_	(CAG)_2_CAAAAG(CAG)CAA(CAG)_8_	(CAG)_2_CAAAAG(CAG)CAA(CAG)_21_
Total CAG repeat		U: 72, L:10	U: 72, L:10	U: 72, L:23

The total length of CAG repeat of all three index patients are all the same, which is 72 repeats of CAG trinucleotides in *ATXN3*. However, the lower allele in patient #3 is 23, different to patient #1 and #2 (Table 5).

## 2 Discussion

This study is the first case series of SCA study in Indonesia. We identified 3 index patients from 3 families that came to us with chief complaints of unsteady walking and gait problems that also appeared in other relatives/family members.

We diagnosed these three index patients with spinocerebellar ataxia type 3 (SCA3). Although onset and clinical signs varies, the clinical findings and progressivity are classic for spinocerebellar ataxia, with unsteady walk as the first sign followed by slurred and scanning speech, dizziness, cranial nerve palsy, ophthalmoplegia, paresis, and numbness [1, 2, 11, 13, 14, 15]. Patient #2, had the earliest onset at 14 years old is considered to be early, onset of SCA3 is commonly in the second to fifth decade [15].

The clinical severity shows a prodromal, mild, and moderate ataxia for patient #1, #2 and #3. Despite the early onset of patient #2, after some physical rehabilitation some function could be maintained, so the severity is not so bad compared to patient #3. Some extracerebellar structural involvement also appears and as it was shown from INAS analysis results (Table 4). Independency of patients showed that only patient #2 has dependency, which was moderately dependent.

Family history showed the pattern of inheritance is autosomal dominant. With no generation skipped and both male and female are affected. Most of SCA is autosomal dominant, only a small number is recessive, yet the number is growing.

After SCA panel analysis, using mostly our *in-house* primer (supplementary table), we found out that all these three index patients have CAG repeats expansion in *ATXN3 (SCA3)*, a gene that encodes *Ataxin3*. SCA3 also known as Machado-Joseph disease, this SCA type is the most prevalent SCA and present with progressive ataxia and plasticity [11].

In our index patients, age of onset was 18 for patient #1, 14 for patient #2 and 27 for patient #3. This is rather early compared to means from a cohort in a study which is 20–50 year-old, mean 37 year old [16], but is still in the range of known onset. This finding happened may be due to some factors including long polyQ repeats. PolyQ repeat plays an important role in patient clinical manifestation, with anticipation is known to be related to onset and severity of the patient. All the index patients have 72 CAG repeats. The clinical symptom is so variable, so some expert classified into 4 subtypes: type 1 early-onset disease with extrapyramidal signs and spasticity but minimal ataxia, type 2 midlife progressive ataxia, type 3 later-onset ataxia accompanied by neuropathy, amyotrophy and loss of reflexes, type 4 parkinsonism with and without ataxia [1, 11, 17, 18]. Based on this clinical classification, all these index patients are classified as subtype 1 of SCA3 [11].

In this study, CAG expansion analysis was performed by using PCR followed by Sanger Sequencing methods. With this method we would be able to analyze the length and the pattern of repeat sequences more precise, compared to other available methods, such as using an internal size marker in the Genescan 500LIZ method [19, 20]. The length of CAG repeat in these three index patients is the same, heterogeneous of 72 CAG repeats with and without GAC sequence interruption as depicted on Table 1. Uninterrupted repeat length vs disease severity relationship has been confirmed, interruption does not significantly change the protein aggregation, however slow down the aggregation rate [21]. An interruption of polyQ repeat by silent (CAA) or missense (CAT) could strongly inflect the effect of the expansion and delay the onset, in other word, onset inversely correlates linearly with the longer uninterrupted CAG repeats. This might have contributed to slow down of aggregation in polyQ interrupted [21].

The length of polyQ repeats inversely correlates with the disease onset, and severity. However this is also affected by other factors. Patient #2 showed the earliest onset among these 3 index patients. All patients have contiguous CAG repeat length above pathogenic threshold [21, 22, 23, 24]. An intermediate repeat allele may form a pathogenic allele via loss of interruption or anticipation (in the next generation) [21]. Threshold should not be seen as a cut off related to toxicity, misfolding, protein aggregation and neuronal cell death, moreover it could accumulate overtime (late onset) [25, 26].

Although the total length of CAG repeats of all three index patients are all the same which is 72, however the age of onset and severity are different. We are still studying these families to know more about the factors behind the difference in the clinical severity and onset between patient #1, #2 and #3, although they have the same number of repeats. These variations of age of onset and severity are also depicted in a study in mainland China which enrolled almost 400 patients [27]. Jiao et al postulates that age is a potential modifier of SCA3 phenotype severity. Further study is needed to evaluate the modulators in our index patients. The connection between arrangements of long and short CAG repeats (i.e. long followed by short, or vice versa), which is not the case to these index patients, to the severity and onset of clinical symptoms are is still unknown. Additionally, patient #3 also has a different length of CAG repeat on the other allele, which is 10 repeats for patient #1, and 23 repeats in patient #3.

Adding to the effort of the neurologist, geneticist, epidemiologist and other scientist on elucidating spinocerebellar ataxia in Indonesia, this study of SCA3 index patient is a very important study to get a comprehension of ataxia epidemiology in Indonesia. Further study to understand more about clinical phenotype, progressivity and the genetic feature of the relatives of this index patient is warranted. An endeavor to devise plans and protocols on how to identify and manage SCA patients in Indonesian or South East Asia or developing countries with its own intricate demography, geography characteristics is possible and indeed a certain necessity.

### 2.1 Patient perspective

"*On behalf of the family, we are relieved that* *the mystery of these clinical features appeared in our family have started to be revealed. These symptoms*, *now diagnosed as spinocerebellar ataxia type 3, was before considered as a curse to our big family, and were not known to us before being evaluated here. We hope after this, although still no definitive treatment available, more of our family members will be evaluated. This endeavour indeed gives hope that we could be helped, together in facing this condition with the doctors.*"

### 2.2 Ethical clearance and informed consent

Ethical clearance was obtained from Universitas Padjadjaran Research Ethics Committee no 0718071150, informed consent was obtained from all respective patients. All related international and privacy protocols have also been obeyed.

## 3 Methods

### 3.1 Tools of ataxia

Phenotyping by clinical evaluation, and neuropsychological studies was performed in the affected subjects at the Department of Neurology, Faculty of Medicine Universitas Padjadjaran/Dr. Hasan Sadikin Central General Hospital, Indonesia. Tools of ataxia that used were classical and well-established, including inventory of non-ataxia signs (INAS) for non-cerebellar signs, which is a classical method to evaluate non-ataxia sign for non-cerebellar sign [28, 29]; scale for the assessment and rating of ataxia (SARA) for ataxia progressivity [30], Montreal cognitive assessment-Indonesian version (MOCA-INA) [31] for cognition and Barthel Index for daily living independency [32].

### 3.2 DNA isolation

DNA isolation was isolated from 3 ml blood using Quick-DNA Miniprep isolation kit (Zymo research) according to manufacturer protocols. DNA concentration was measured using Nanodrop^TM2000^.

### 3.3 SCA PolyQ repeat panel analysis

PolyQ repeat expansion analysis for SCA 1, 2, 3, and 6 were performed using polymerase chain reaction (PCR) method followed by Sanger Sequencing [7]. 50–100 ng DNA, 25 μl of MyTaq™HS Red Mix (Bioline), 1 μl of each 10 μM forward and reverse primers and MQ water (Millipore) was add to have total reaction volume of 50 μl. Primers used in this study mostly were in-house design using Primer 3 v 0.4.0 (supplementary table) [33] except for SCA6 which was from Zhuchenko, et. al, 1997 [34]. PCR was performed using touch-down PCR (TD-PCR) method with the annealing temperature ranging from 65 °C - 55 °C. The first 10 cycle the annealing temperature was reduced by 1 °C/cycle until it reached 56 °C. The annealing temperature for last 25 cycles was 55 °C. PCR products were run on Agarose gel (0.8%) electrophoresis to check the present of the upper (mutant allele) and lower (wild type allele) PCR bands. Both alleles were gel-purified and sequenced using forward and reverse primers on ABI-3500 (Applied Bio System).

### 3.4 CARE checklist

To ensure a standard and concise method of writing a case report, CARE checklist is used in writing this article.

## Declarations

### Author contribution statement

All authors listed have significantly contributed to the investigation, development and writing of this article.

### Funding statement

This work was supported by Fundamental Research Fund no. 1827/UN6.3.1/LT/2020 for Tri Hanggono Achmad from the Directorate General of Higher Education, the Ministry of Education and Culture/ National Research and Innovation Agency (BRIN), the Republic of Indonesia.

### Data availability statement

The authors are unable or have chosen not to specify which data has been used.

### Declaration of interests statement

The authors declare no conflict of interest.

### Additional information

No additional information is available for this paper.

## Appendix A Supplementary data

Supplement table - Various primers for sequencing SCAs Supplement table - Various primers for sequencing SCAs
