# Supplementary material for: Clinical and genetic profile in index patients with spinocerebellar ataxia type 3 in Indonesia: case report
Source: Heliyon. 2021 Jul 7;7(7):e07519. doi: 10.1016/j.heliyon.2021.e07519 (PMC8319015; doi:10.1016/j.heliyon.2021.e07519)
Supplement: Supplement table - Various primers for sequencing SCAs [file mmc1.pdf]

Supplement to HELIYON doi:10.1016/j.heliyon.2021.e07519
CLINICAL AND GENETIC PROFILE IN INDEX PATIENTS WITH SPINOCEREBELLAR ATAXIA TYPE 3 IN INDONESIA: CASE REPORT
Siti Aminah, Fathul Huda, Uni Gamayani, et al.

Supplementary table. Various in house and reference primers for sequencing Spinocerebellar ataxias

No	Primer Name	Sequence 5'→ 3'	product (bp)	Tm (°C)
1	SCA1_HM_F	GAGGCCTATTCCACTCTGC	314	57,4
2	SCA1_HM_R	TGTGGGATCATCGTCTGG		58,9
3	SCA2_HM_F	GTTCCGGCGTCTCCTTGG	336	68,5
4	SCA2_HM_R	ACACCCACCTGCCCAGC		67,9
5	SCA3_HM_F	TTCCTAAGATCAGCACTTCC	295	54,57
6	SCA3_HM_R	CAAGTGCTCCTGAACTGG		55,14
7	SCA6_S-5_F1*	CACGTGTCCTATTCCCTGTGATCC	141	68,7
8	SCA6_S-5_R1*	TGGGTACCTCCGAGGGCCGCTGGTG		79
9	SCA7_HM_F	TAGGAGCGGAAAGAATGTCG	307	60,34
10	SCA7_HM_R	CACAGATTCCACGACTGTCC		59,1

bp: base pair; F: forward sequence; HM: home made/ inhouse primer; R: reverse sequence; Tm: primer
* sequence acquired from Zhuchenko et al., Nature Genet., 15, 62-69 (1997)
